# The Impact of Coffee and Caffeine on Multiple Sclerosis Compared to Other Neurodegenerative Diseases

**DOI:** 10.3389/fnut.2018.00133

**Published:** 2018-12-21

**Authors:** Lena Herden, Robert Weissert

**Affiliations:** Department of Neurology, University of Regensburg, Regensburg, Germany

**Keywords:** neuroinflammation, coffee, caffeine, adenosine, multiple sclerosis, Parkinson's disease, amyotrophic lateral sclerosis, neuroprotection

## Abstract

**Background:** The literature concerning the effect of coffee and caffeine on Multiple Sclerosis (MS) with focus on fatigue is investigated in this review. Potentially clinically relevant effects were also assessed in studies concerning comparable neurodegenerative diseases, such as Parkinson's disease (PD) and amyotrophic lateral sclerosis (ALS). Since the existing studies obtained very inconclusive results, we systematically reviewed these studies to summarize the evidence on the possible effects of coffee and caffeine on those disease entities. Previous studies suggested that coffee and caffeine intake is associated with a reduced risk of developing MS and other neurological diseases.

**Methods:** The PubMed database was searched using the keywords “coffee” OR “caffeine” in combination with keywords for each of the different diseases. Besides the keyword search, we included studies by reference list search. Studies on the effects of coffee and caffeine on the single neurological diseases were included for this review. A total of 51 articles met our inclusion criteria. The reviewed articles assessed the impact of coffee and caffeine on the susceptibility for neurological diseases, as well as the effect of coffee and caffeine on disease progression and possible symptomatic effects like on performance enhancement.

**Results:** Higher intake of coffee and caffeine was associated with a lower risk of developing PD. In some of the MS studies there, is evidence for a similar effect and experimental studies confirmed the positive impact. Interestingly in MS coffee and caffeine may have a stronger impact on disease course compared to effects on disease susceptibility. In ALS no such beneficial effect could be observed in the clinical and experimental studies.

**Conclusion:** This literature assessment revealed that coffee and especially caffeine could have a preventative role in the development of several neurodegenerative diseases if provided in comparatively high doses. The systematic assessment indicates that coffee and caffeine intake must not be considered as a health risk. Additional clinical studies are needed to fully understand how far coffee and caffeine intake should be considered as a potential therapeutic approach for certain disease entities and conditions.

## Introduction

### Multiple Sclerosis

Multiple Sclerosis (MS) is a chronic autoimmune disease of the central nervous system (CNS) that can affect individuals in all ages. Worldwide an estimated amount of 2.5 million people suffers from MS. It is assumed that T cells specifically identify and attack components of myelin sheaths in the CNS. The cause of MS has not been discovered yet, but it has been reported that genetic, as well as environmental factors contribute to its precipitation (1, 2). In how far those factors act in combination is presently investigated in different laboratories. MS leads to inflammation and demyelination, causing lesions in the white and gray matter. The resulting conductance reduction or/and block, axonal damage, and loss of neurons are responsible for the symptoms in MS. Such symptoms include numbness, vision disturbances, brain stem symptoms, bladder dysfunction, paresis, ataxia, and a slowly developing cognitive disability, depending on where the lesions are located.

#### MS Disease Course and Fatigue

MS is a disease with various disease courses. The majority of the patients first develop a relapsing-remitting type of MS (RRMS) which can transform into a secondary chronic progressive type (SPMS) over time (3). About 15% of patients develop a primary progressive MS (PPMS).

There are also non-physical symptoms with fatigue the most relevant. Fatigue is an extreme exhaustion that overcomes patients without premonition. There exist various definitions for fatigue, most important is the fact that sleepiness and fatigue do not have the same meaning (4). In 2007 a group of neurologists closely investigated fatigue on patients with MS to finally set one definition as standard. They conclude, that “fatigue is defined, as reversible, motor, and cognitive impairment with reduced motivation and desire to rest, either appearing spontaneously or brought on by mental or physical activity, humidity, acute infection, and food ingestion. It is relieved by daytime sleep or rest without sleep. It can occur at any time but is usually worse in the afternoon. In MS, fatigue can be daily, has usually been present for years and has greater severity than any premorbid fatigue” (5). Fatigue is more than just the feeling of being tired. Patients suffering from fatigue often cannot manage a normal day without taking breaks. Especially fatigue has a strong impact on the working capacity of patients (4). More than 70% of the patients with MS are reporting about symptoms of fatigue (6). Studies have shown that 14% of patients with MS consider fatigue their worst problem and 55% as one of their worst problems (7). It is also one of the main reasons for unemployment and early retirement (8–10). Patients with multiple sclerosis-related fatigue (MSRF) have a significantly increased mental and physical disability and a lower quality of life (11).

Although fatigue is such a profound symptom, the mechanisms behind its onset have not been identified yet. A recent study showed that there is possibly a correlation between fatigue and structural damage in the brain (12). Currently, there is no approved medical therapy for fatigue available. Several substances have been evaluated regarding its influence on fatigue. Even meta-analysis about various pharmacological therapies could not give evidence for an effective treatment (13). There exist single studies indicating that modafinil (14) and amantadine (15) might have a positive effect on the condition of patients, but in further meta-analysis no significant impact could be demonstrated. Due to missing comparative studies between several therapeutic approaches, there still exists no first-line therapy for fatigue.

A recent meta-analysis has shown that psychological interventions, like cognitive behavioral therapy, relaxation exercises, and mindfulness interventions could significantly reduce fatigue levels (16). Thus, at trying to treat fatigue, currently, it is most important to inform patients about fatigue. It is advised to ask for sleeping disorders, to check the current medications for tiring side effects, and explain the value of sports and physical activity during the day for preventing or tempering fatigue (13, 17). However, the treatment options are still limited.

Since coffee and caffeine showed a beneficial effect on daytime somnolence in Parkinson's disease (PD) (18) a comparable positive effect in MS might be assumed. The effects of coffee and caffeine on MS-related fatigue have not been investigated yet. Still few studies exist that assessed the effect of coffee and caffeine on disease susceptibility and course.

Therefore, in this review we focused on summarizing the possible effects of coffee and caffeine in MS. Since literature cannot present enough data to provide a clear statement, we also reviewed studies concerning its effects on PD and ALS with the intention to obtain an overview and a better understanding of how coffee and caffeine might act in the CNS.

### Coffee

#### The History of Coffee and Caffeine as a Medical Treatment

The use of caffeine as a medical treatment started off slowly in the early nineteenth century, when 1,819 the German chemist Friedlieb Ferdinand Runge isolated caffeine for the first time. Half a century later, in 1886 the first version of caffeine containing Coca-Cola was sold as a patent medicine for headache (19). Thereafter the interest in medical effects of caffeine and coffee consumption has increased rapidly.

Even if coffee was introduced in Europe only a few 100 years ago (Box 1), it has reached an important position in our culture. By now coffee is one of the most popular beverages all over the world. Not just because of its taste, particularly the caffeine content leads to its enormous popularity. In the last years, the way of consuming coffee has experienced a massive transformation. Especially the popularity in younger adults has risen in the last decades. People go to coffee shops to have a coffee every day, meeting friends, taking a break or just enjoy a cup of cappuccino. Most people from all cultures consume coffee frequently if not daily.

Box 1 **The history of coffee (20–23).**

The knowledge about coffee, in the way we consume it presumably developed in the 15^th^ century in the region of Yemen. Earlier references appeared only in legends, suggesting a 9^th^ century origin in Ethiopia and Yemen. Interestingly, at that time the green coffee beans were just eaten raw. In the 16^th^ century, the coffee bean spread to the Middle East, South India, Persia, Turkey, and Northern Africa and has found its way into Europe via Italy. The former Republic of Venice kept a vibrant trade with Muslims from Northern Africa. In the early 17^th^ century coffee appeared in Venice, in which a short time after the first European coffee house opened. Once in Europe, coffee gained increasingly in popularity and spread so fast, that in 1652 the first London coffee house opened.

#### Composition

Coffee consists of more than 1,000 ingredients (Table 1), including carbohydrates, lipids, vitamins, minerals, and alkaloids (32), depending on the variety and the further processing. Coffee is one of the most important sources of chlorogenic acids (33). There have been studies, showing the antioxidative effect of chlorogenic acids, and caffeic acids *in vitro* (34), yet there are no results showing this effect *in vivo*. This may be due to the massive metabolism of the acids in the human body. From all the ingredients, caffeine is the most investigated one. Its psychostimulant effect has been reported as well as its positive short-term effect on attention and mental condition like cognition and memory (35). Coffee is one of the most essential sources of caffeine, but also soft drinks and energy drinks cover up a significant part of the caffeine intake, especially for children (36) (Table 2).

**Table 1:** Average chemical composition of coffee Arabica beans in % of the dry matter, before, and after roasting (24–31).

**Ingredient**		**Background**	**Raw, green coffee beans in %**	**Roasted coffee beans in %**
Caffeine		Alkaloid, psychoactive stimulant of the central nervous system	**1.2**	**1.3**
Carbohydrates		Small amounts of simple carbohydrates such as fructose, glucose, mannose, arabinose, and rhamnose and oligosaccharides such as raffinose and stachyose have been identified in green coffee	**46.0**	**35.0**
	*Sucrose*	*Important for coffee flavor and quality*	*8.0*	*0*
	*Polysaccharides*	*The main polysaccharides in coffee are galactomannan and arabinogalactan. Both of them are soluble*.	*35–44*	*32.0*
	*Lignin*	*A class of complex organic polymers, structural material in the support tissue of plants, important for the formation of cell walls*	*3.0*	*3.0*
Chlorogenic acids		An Ester of the caffeic acid, found in many plants. Chlorogenic acids are assumed to cause gastrointestinal discomfort at some people with higher coffee consumption. They can cause a slight reduction in blood pressure and have been investigated concerning an anti-inflammatory effect. They have an antioxidant effect	**6.5**	**2.5**
Lipids		Mostly coffee oil (triglycerides with unsapoifiables and sterols/tocopherols) and diterpenes (cafestol and kahweol)	**16.0**	**17.0**
Minerals		Mostly potassium and phosphorus. Also, sodium, magnesium, calcium, and sulfur	**4.2**	**4.5**
Products of caramelization and condensation		Substances, which develop through roasting, influencing the color, and aroma of coffee		**25.5**
Proteins		Peptides and free amino acids, vital for the flavor of coffee	**11.0**	**7.5**
Trigonelline		Alkaloid, found in many plants. Roasting metabolizes part of it to niacin (VitB3)	**1.0–1.2**	**0.5–1.2**

**Table 2:** Beverages containing caffeine with data about caffeine content (37–40).

	**Caffeine per**	**Serving**	**Caffeine per**
	**100 ml [mg]**	**size [ml]**	**serving [mg]**
Brewed coffee	60–100	150	90–150
Espresso	100–150	30	30–50
Instant Coffee	27–72	150	40–108
Decaffeinated	1–3	150	2–5
Tea	6–22	250	15–55
Iced tea	6–10	250	15–25
Coca cola	10	250	25
Diet coke	13	250	33
Decaffeinated coke	0	250	0
Energy drinks monster	34	250	85
Red bull	34	250	85
Energy Shots e.g., 5-h-energy	333	60	200
Chocolate Milk beverage	1–3	250	2–7

#### Pharmacokinetics

The amount of caffeine in coffee varies among different sorts. It has been shown, that there are diverse effects on the CNS, comparing coffee Arabica to coffee Robusta (41). The caffeine contained in coffee is absorbed very fast compared to other sources (42). It is completely absorbed within 45 min after oral ingestion. It reaches its highest plasma concentration after 20–30 min, and due to its hydrophobic structure, it can pass the blood-brain barrier (BBB) easily to have its full effect on the CNS (43, 44).

### Caffeine

Caffeine (1, 3, 7-trimethylxanthine) belongs to the purines and its main effect is as a psychostimulant in the CNS. The stimulant effects of caffeine are due to its ability to decrease the transmission of adenosine in the various regions of the brain (45).

#### Adenosine Receptors

Adenosine, which structure is very similar to caffeine, is considered a neuromodulator. It plays a critical role in several biochemical processes, generally inhibiting excitability in the CNS through different pathways, activated by different types of adenosine receptors. There exist various types of adenosine receptors: A1, A2a, A2b, and A3. They can be found in different cells in the CNS, such as astrocytes, microglia, and neuronal cells of the striatum, and the spinal cord. Also in immune cells and endothelial cells adenosine receptors are expressed (46). Caffeine stimulates the CNS, as well as improves cognitive function, reaction time, wakefulness, concentration, and motor coordination (47).

The binding of adenosine to A1 and A3 receptors lead to inhibition of the adenylyl cyclase through activation of the inhibitory G-protein. Thus, less cyclic AMP (cAMP) is available, causing a reduced activation of the protein kinase A (PKA). The inactivated PKA causes a reduced flow of calcium into the cell. In contrary, when adenosine binds to A2a or A2b receptors it activates a stimulatory G-protein inducing the opposite effects leading to an increase of intracellular calcium (48).

Caffeine acts as an antagonist on adenosine receptors. At 300 mg of intake (which is equivalent to 2–3 cups [150 ml each] of coffee), it has its highest affinity for A1 and A2a receptors (49). The A1, as well as the A2a receptors, are both G-protein-coupled but lead to opposite effects. When adenosine is binding to A2b receptors in cells in which the A1 receptor is also expressed, it inhibits A1 receptors to bind adenosine and increases calcium influx (50). The A1 receptor can be found all over the CNS, whereas A2a receptors were found concentrated in dopamine rich areas of the brain (51, 52). The blocking of these receptors downregulates the release of dopamine, noradrenaline, and glutamate (53). Solely blocking of the A2a receptor reduces direct calcium entry into neurons and decreases inflammatory reactivity of microglia (54). Glutamate is a neurotransmitter which causes cytosolic calcium release leading to a more severe inflammatory response (55, 56). Upregulation of A1 receptors has an inverse effect on the production of pro-inflammatory cytokines, in particular, tumor necrosis factor alpha (TNF-α) (57). In conclusion, caffeine leads to an anti-inflammatory effect, mainly via the antagonism of the A2b receptor and there is also evidence that the A1 receptor might contribute to this effect.

#### Further Actions in the Brain

The total of the effects of caffeine on the cellular level is incompletely understood. It modifies the synaptic and ectopic vesicle release (58). Moreover, it interferes with GABA-A receptors (59). The upregulation of GABA receptors is due to the decrease of GABA concentration induced by caffeine. GABA normally modulates our sensation of alertness and tiredness and helps us relax. In the situation of reduced GABA concentrations in the CNS, the body feels more active, and the motor activity is higher. Additionally, it leads to a non-selective competitive inhibition of phosphodiesterase (PDE) and an activation of the ryanodine receptor (60). Inhibitors of the PDE simultaneously inhibit the inactivation of second messengers, like cAMP. Due to this, the amplification of intracellular signals through second messenger is extended. Because of the activation of ryanodine receptors, a calcium releasing channel on the endoplasmic reticulum (ER), caffeine and especially its metabolite paraxanthine can induce calcium release from the sarcoplasmic reticulum and inhibit its reuptake (61). Caffeine can reduce cerebral blood flow (62). However, some of the described effects on cellular function could only be observed *in vitro*. *In vivo*, much higher levels of caffeine are required (36, 63). The plasma concentrations required to have such effects in humans cannot be reached by coffee intake (43).

All the therapeutic and adverse effects differ immensely depending on whether the long-term or acute effect of coffee consumption is considered. Chronic caffeine administration may be responsible for the possible neuroprotective effect of caffeine, due to its increased adenosine plasma concentration (64). Whereas, the acute effects of caffeine intake lead to the well-known psychic activation.

#### Actions of Caffeine on the Body

In addition to the above described effects on the CNS, caffeine does have effects on the whole body. Caffeine leads to a bronchodilation, a peripheral vasodilation, and due to its positive inotropic effect increases the contractility and efficiency of the heart (61). It stimulates digestion by promoting peristalsis and has diuretic effects on kidney (65). Consequently, in the last years, the consumption of coffee has been discussed controversially. It is known that caffeine (i.e., 150 ml coffee/cup) consumption has short-term effects on blood pressure in individuals with normotensive or hypertensive pressure (66). Regardless, studies examining the long-term effects on blood pressure in habitual coffee drinkers did not yield significant results (67). A recent meta-analysis did not demonstrate clinical evidence for effects of long-term coffee consumption on blood pressure (68). Additional studies as well did not show evidence of health risks mediated by caffeine but found evidence of potential benefits. Taken together the results indicate that a coffee consumption of up to four cups of coffee per day can be considered completely healthy (69). In some of those studies, an association between high coffee intake and an unhealthy lifestyle was assumed, including behaviors like smoking or less physical activity which may have caused the wrong assumptions (67).

## Methods

This systematic review was performed in accordance with the guideline of the Preferred Reporting Items for Systematic Reviews and Meta-Analysis (PRISMA) statement (70).

### Literature Research

Studies were identified by searching the PubMed database and scanning reference lists of articles. Database searches were conducted between 15th of May 2018 and the 18th of June 2018 by both authors separately. The first literature search was done by LH and for revision by RW. There was no date or language restriction in the search strategy. For the studies concerning MS we searched by the terms “coffee” “OR” “caffeine” “AND” “multiple sclerosis.” We obtained 38 articles and after scanning the abstract we selected six studies meeting the criteria for this review. We repeated this for the studies concerning ALS, and by searching for “coffee” “OR” “caffeine” “AND” “amyotrophic lateral sclerosis” “OR” “ALS.” We obtained 33 articles, of which only four met our review criteria. For studies concerning PD, we used the terms “coffee” “AND” “Parkinson^*^” and obtained 219 articles. We included 34 of those for our review. Additionally, we included seven articles which we found by scanning reference lists, leading to a total of 41 PD studies.

### Study Selection

Due to few existing studies, we included prospective and retrospective clinical and epidemiological studies as well as animal and *in vitro* studies that dealt with the possible mechanisms of action. Therefore, we categorized the studies by study design for the revision.

In total 281 articles were obtained from the PubMed database search. For MS 38 articles were obtained by database search. 25 of those were excluded because they also did not investigate the effect of coffee or caffeine or/and did not deal with MS. Three articles were excluded for not presenting all data of the study or reviewing other studies insufficiently. There was no full-text available for four articles. Therefore, the six remaining studies were included for the review. The 210 articles obtained for PD were reviewed. One hundred and forty three articles did not meet our inclusion criteria because they did not investigate PD or/and did not investigate the effect of coffee or caffeine. Another 16 articles were excluded because of not meeting high quality, like just reviewing existing studies or reviewing articles which did not present any outcome data. Lastly, 17 articles were rejected because no full text was provided. Through the 34 included studies we obtained seven additional articles by scanning the reference lists, which resulted in a total of 41 included articles. Equally, the 33 articles on ALS obtained by database search were examined. Twenty six of the search results were articles, which again did not investigate either the effect of coffee or caffeine, or the effect on ALS. One article reviewed literature and for two articles no full-text was provided. In total, four articles on ALS were included. A flowchart about the research strategy was created (Supplementary Figure 1). The total of 51 selected studies are summarized in Table 3 and Supplementary Tables 1–3.

**Table 3:** Studies evaluating the effect of coffee on multiple Sclerosis and EAE.

	**References**	**Study design**	**Cases**	**Findings**
Clinical trial	Hedström et al. (Sweden, US) (71)	Comparison of two population representative case-control studies based on retrospective data collection	1,620 cases and 2,788 controls in the Swedish study and 1,159 cases and 1,172 controls in the US study Age 16–70	The risk of MS is substantially reduced among those who reported a high consumption of coffee, exceeding 900 ml daily OR 0.70 (95% CI 0.49–0.99 in the Swedish study) and OR 0.69 (95% CI 0.5–0.96 in the US study).
	Massa et al. (US) (72)	Statistical analysis, concerning the intake of alcohol and caffeine were examined in relation to the risk of MS in two large cohorts of women	Nurses' Health study (NHS) 92.275 women followed for 24 years and NHSII 95,051 women followed for 14 years 282 MS cases during the follow up Age 30–55 and 25–42	No significant association was found between coffee or caffeine intake and the risk of MS. The evaluation of caffeinated coffee vs. decaffeinated coffee also yielded no results.
	Ponsonby et al. (Australia) (73)	Case-control study assessed life-style factors, like smoking alcohol or coffee intake and physical activity, prior to a first clinical demyelinating event	Cases *n* = 282, Controls without CNS Demyelination *n* = 558, matched in age and sex and study region Age 18–59	No significant results concerning an effect of coffee could be shown. Still the case groups were much more likely to a coffee intake of five or more cups/day in the last year.
	D'hooghe et al. (Belgium, the Netherlands) (74)	Cross sectional survey amongst individuals with MS, with time to EDSS 6 as outcome measure	1,372 persons with definite MS were analyzed Age 17–89	An extension by 4 years for the time to reach EDSS 6 since birth was demonstrated in the group with daily coffee intake, compared to the group with individuals who never drank coffee (*p* = 0.002)
	Pekmezovic et al. (Serbia) (75)	Case control study evaluating association between the risk of MS and lifestyle factors like cigarette smoking and coffee, and alcohol consumption	*n* = 210 cases with definite MS, n = 210 controls, matched in age and sex Mean Age 33,6 ± 10,2	Coffee consumption was significantly more frequent in the MS-group but was not considered a risk factor. A dose-response relationship was shown between risk of MS and both, number of cups/day (*p* = 0.039) and duration of coffee intake in years (*p* = 0.01)
Animal Model	Chen et al. (China) (76)	The attenuation of guinea pig spinal cord homogenate induced pathology by chronic caffeine treatment was observed at doses of 10 and 30 mg/kg and during both peak and recovery phases of EAE	126 females, EAE induced Wistar rats	Caffeine decreases the incidence of EAE and attenuates EAE pathology at behavioral, histological, and neurochemical levels.Chronic treatment with caffeine up-regulated A1 receptor and TGF-β mRNAs and suppressed interferon-γ mRNA in EAE

### Data Extraction

The data extracted from the eligible studies included: authors, year, and country of publication, study design (type of intervention and main aim of the study as the primary outcome), intervention details (like doses of coffee or caffeine), duration (if given also time of follow-up period), population (sample size, mean age, gender distribution—if mentioned in the article), and the findings of the single studies.

## Results

### Coffee and MS

The six studies regarding MS and coffee or caffeine effects that fitted the inclusion criteria for detailed evaluation in this review, showed a high heterogeneity in their designs (Table 3). The limited number of studies assessing the effect of coffee or caffeine on MS did not allow performing a meaningful meta-analysis. Still some conclusions can be drawn.

Two matched case-control studies (*n* = 210 and *n* = 282) of the total of four studies, which assessed an effect on MS susceptibility, could demonstrate an increased intake of coffee in patients with MS, compared to the healthy controls (73, 75). Still, both studies could not determine coffee as a significant risk factor for MS, due to their very inconclusive findings.

Another study found no significant association between coffee and the risk for MS, neither positive nor negative (72). Two hundred and eighty two cases of MS out of a total of *n* = 187,326 women were found. A more recent comparison of two studies from 2015 (in total *n* = 2,779 cases and *n* = 3,960 controls) observed a significant association between high consumption of coffee (>900 ml daily) and a decreased risk for MS (OR 0.70, *p* = 0.01 and OR 0.69 and *p* = 0.05 in the individual studies) (71).

A cross-sectional survey from 2011 (*n* = 1,372) showed that coffee consumption does have a positive effect on the disease course and progression, at least in the relapsing form of MS (74). The authors set the time for reaching EDSS (Expanded Disability Status Scale) step 6 as an outcome measure and compared groups with different intake levels of coffee. They discovered a significant extension of that time by 4 years comparing the daily coffee drinkers to the never coffee drinkers (*p* = 0.002).

An experimental study was performed in 2009 to evaluate if chronic caffeine treatment has any neuroprotective effects on the course of an animal model of MS, namely experimental autoimmune encephalomyelitis (EAE) in rats (76). The used EAE model is characterized by extensive tissue inflammation and a chronic disease course. The incidence of EAE decreased within the caffeine treated rats (*n* = 126) and an attenuation of the disease on behavioral, neurochemical, and histological levels (*p* < 0.005 compared to rats, who received only water) was demonstrated.

#### Coffee and Amyotrophic Lateral Sclerosis

Amyotrophic lateral sclerosis (ALS) is an incurable disease characterized by progressive degeneration of motor neurons in spinal cord and motor cortex (77). Only a few studies have investigated the relationship between coffee or caffeine and the effects on patients with ALS (Supplementary Table 1). While a recent meta-analysis of five large cohort studies (total *n* = 1,010,000 with *n* = 1,279 cases of ALS) has not found any association between coffee intake and the risk of ALS (78), a prior case-control study (*n* = 377 cases and *n* = 754 controls) did find an inverse effect of coffee consumption and the risk of ALS (79). The authors compared the ALS cases to neurological and non-neurological hospital controls. They reported a lower risk for ALS among ever coffee drinkers (intake >1 cup/day for at least 6 months during lifetime), compared with never coffee drinkers (*OR* = 0.6, 95% *CI* = 0.4–0.8).

A preceding prospective study from 2008 (80) assessed the effect of various dietary habits on ALS and found no significant association between caffeinated coffee and the risk of developing ALS. Interestingly the authors observed a significant relationship between a high intake of decaffeinated coffee (≥4 cups/day) and the risk of ALS (*p* = 0.01).

There exist only one experimental study which examined the presumed neuroprotective effect of chronic caffeine administration in an animal model of ALS (81). In a transgenic model of ALS, SOD1(G93A) mice (SOD, superoxide dismutase 1) that received caffeine compared to controls receiving water had lower body weight, reduced motor performance, earlier disease onset, and reduced survival times. Specifically, caffeine treatment accelerated disease progression and shortened survival time by an average of 12 days (*p* = 0.01).

#### Coffee and Parkinson's Disease

The association between coffee and caffeine and a reduced risk of developing PD has been investigated in the last 50 years. Although the existing studies differ in their results, there is evidence for possible neuroprotective effects of caffeine in PD. The combined relative risk in a recent meta-analysis was 0.67 (95% CI 0.58, 0.76) (82). Therefore, we reviewed the published studies (*n* = 40) from 1968 until today (Supplementary Tables 2, 3).

Five of the studies evaluated the effect of coffee on the progression or the symptomatic enhancement of PD (18, 83–86). Results were contradictory but for some evaluated symptoms, coffee showed a beneficial effect. Caffeine improved the “total akinesia” type of freezing of gait (FOG) in PD. A prospective study (*n* = 16) from 2007 could demonstrate an improvement of the total akinesia for a certain time period. Interestingly the beneficial effect disappeared after a few months, although the effect could be completely restored after a 2-week caffeine withdrawal (86).

An analysis, evaluating the data of two studies (total *n* = 413 PD cases) concerning the rate of progression and the influence of caffeine could not find any significant effects (85).

Two prospective placebo-controlled trials from 2012 (*n* = 61) and 2017 (*n* = 121) did not observe a strong beneficial effect of coffee concerning motor function. A small improvement by 4.7 points (*p* < 0.05) on Unified Parkinson's Disease Rating Scale (UPDRS) was seen (83, 84).

However, somnolence improved modestly in PD patients as seen by reduction of sleepiness documented with the Epworth Sleepiness Scale (ESS) (83). After a 6-week caffeine disposal, ESS was reduced by 1.71 compared to the placebo group. Still, this study provides evidence that a 6-week caffeine treatment does not improve daytime sleepiness significantly in patients with PD. In contrast a recent low-powered prospective study showed that espresso intake had a significant effect on daytime somnolence in PD (18).

The susceptibility of PD and the possible protective effects of coffee have been examined closely over the last decades. Nine prospective cohort studies were performed to evaluate the effect of coffee and caffeine (87–95). All of those found evidence for a significant beneficial effect of coffee consumption on the risk of developing PD.

The most recent prospective cohort study (*n* = 539,222, participants were recruited from the Cancer Prevention Study II and filled-out a questionnaire about dietary habits) found an inverse association between coffee consumption and the risk of developing PD (*RR* = 0.43, 95% *CI* = 0.26–0.71, *p* ≤ 0.002) for caffeine intake at 477 mg/day (3–4 cups a 150 ml) compared to a caffeine intake of 9,2 mg/day in men (92). For women the effect was slightly smaller [RR 0.61 (95% CI: 0.34–1.09; *p* = 0.05)]. Among women, the beneficial effect was stronger among never users of hormone replacement therapy (*RR* = 0.32) than among ever users (*RR* = 0.81, p-interaction = 0.15). Interestingly four of these nine studies found a stronger effect in men compared to women. Several studies came to the same result that women who had never taken postmenopausal estrogen do have an increased risk for developing PD when consuming coffee (91, 92).

Previous case-control studies (96–111) suggest that coffee consumption is inversely associated with the risk of PD.

Two case-control studies (*n* = 812 and *n* = 1,208) investigated the genetic influence on how coffee can affect the risk of PD. No significant association between the ADORA2A, the CYP1A2 gene, and the effect of coffee on PD could be shown (98). The second study assessed the impact of coffee on PD patients with high genetic risk (LRRK2 R1628P variant) compared to those with lower genetic risk for PD. Caffeine intake significantly reduced the risk of PD much more in those patients with high genetic susceptibility (R1628P variant) compared to those with low genetic susceptibility. Patients with high genetic risk who did not take caffeine had 15 times higher risk of PD compared to patients with low genetic risk with caffeine intake. About 70% of this risk increase is due to the interaction of R1628P and no caffeine intake (96).

Retrospective evaluation showed a delay of PD onset by 4.8 years for participants who drank three or more cups of coffee daily (112).

PD is a disease mainly characterized by the reduction of the dopaminergic neurons of substantia nigra due to a neurodegenerative process (113). The experimental studies in animal models that have been reported so far support the role of adenosine receptor antagonists such as caffeine on PD.

The first study from 2001 demonstrated that caffeine in equivalent doses to human intake could attenuate the dopaminergic degeneration of anatomical and functional markers in the MPTP (1-methyl-4-phenyl-1,2,3,6-tetrahydropyridine) experimental model in mice (*n* = 18). (114) MPTP is a neurotoxin, which can induce striatal dopaminergic depletion (115). The authors additionally discovered that A2a receptor-specific antagonists, in contrast to A1 receptor-antagonists, could mimic the neuroprotective effects of caffeine. In 2006, after epidemiological studies in humans provided data of estrogens, which could attenuate the beneficial effect of caffeine in women, this finding was confirmed in the MPTP mouse model of PD (*n* = 22) (116).

In a similar model, the effect of a caffeine administration, 30 min prior to an eight-day MPTP treatment was evaluated (117). The caffeine-treated mice showed less neuronal damage, better paw grip strength, and motor function. In another model of PD, caffeine had a comparable protective effect (118). In the used 6-OHDA (6-hydroxydopamine) model of PD in rats, the injection of OHDA in the brain parenchyma or ventricles leads to noradrenaline-, adrenaline-, and dopamine depletion (*n* = 72) (119). MPP+ (methyl-4-phenylpyridinium) is a neurotoxin, which is infused into one of the lateral ventricles of rats over a time course of 28 days (*n* = 5 per group) (56). The ensuing ipsilateral dopaminergic neuron loss could be prevented when caffeine was given before, as well as arrested when given in drinking water after the injection.

To investigate the *in vitro* cytoprotective mechanisms of caffeine, human dopaminergic neuroblastoma SH-SY5Y cells were used. Caffeine prevented apoptotic cell-death after serum/retinoic acid depletion, MPP+, rotenone, and 6-OHDA applications in a dose-dependent relationship. Additionally, it reduced an induced caspase-3 activity, which suggests that caffeine‘s protective effect is due to the activation of the phosphatidyl-inositol-3-kinase (PI3K)/Akt pathways (120). This pathway is not completely understood yet, nevertheless it is evident that it is involved in inhibition of neuronal apoptosis (121).

## Discussion

Caffeine as a non-selective antagonist of adenosine receptors with its neuroprotective effects as well as the general impact of coffee on chronic and autoimmune diseases have recently been investigated very closely. The precise mechanism underlying caffeine's neuroprotective effect has not been fully understood, yet a beneficial effect could be observed in various disease entities and conditions.

The current state of knowledge permits the conclusion that coffee and caffeine intake in moderation must not be considered as a health risk. Since most people drink coffee for pleasure and because of its taste, a possible health benefit would be an added value.

The existing epidemiological studies lead to the conclusion, that coffee and especially caffeine could have a preventative role in the development of several neurodegenerative diseases. However, recent data in animal models suggest that caffeine may also have therapeutic effects on patients already diagnosed with a certain disease entity. Further clinical studies are needed to fully understand how and in how far coffee and caffeine intake should be regarded as a potential therapeutic approach.

The included studies did have limitations. Possibly in some of the studies, no generalized conclusion should be drawn because of the used inclusion criteria. For example in the Nurses' Health Study I and II only women at a quite similar age were included (72). Another limitation for our review is the situation that not all the assessed studies specified the exact amount of coffee or caffeine intake in the in investigated groups of patients. Some of the studies only counted the number of cups per day which may vary in size and dose. The single compliance in the different studies is mainly unclear, as well as in how far the subjectively stated amount of coffee intake can be trusted. Nevertheless, we tried to secure quality by only including original peer-reviewed articles.

With most of the studies assessing the influence of different dietary habits, it would be interesting to evaluate the effect of coffee or caffeine in isolation more closely. Examining several dietary habits at once raises the question, whether the other habits must be looked at as confounders and possibly must be considered in the results. It is not clear yet, in how far this may modify the results for the effect of coffee and caffeine intake. Additional studies, more specified on the effect of coffee and caffeine should rule out the possible effect of confounders.

At this point it should be mentioned, that coffee consumption should not be equated with caffeine intake. There are far more ingredients in a cup of coffee than just caffeine. Even if caffeine is the most investigated one, other contents have been observed to have a not insignificant effect on the body as well (Table 1). Attention should be given to the various contents and how they may work in combination. Additionally, the relaxing effect of taking a coffee break may play a more pronounced role than assumed. Coffee can be used for a more structured daily routine and the assessment of the psychological impact of a hot cup of coffee would be interesting for research. Regarding this, studies should aim to compare the effect of caffeine, e.g., as caffeine pills with the effect of the same amount of caffeine in coffee.

Interestingly this review revealed that caffeine may have beneficial effects in MS and PD but not in ALS. None of the epidemiological or experimental studies have found a positive impact of caffeine intake on susceptibility or disease course of ALS. In additional prospective clinical studies, it would be interesting to see, if an effect comparable to MS and PD can be found. In PD the results suggest a potential motor benefit. Larger long-term studies would be desirable. The studies in PD on the possible risk-reducing effect of coffee vary in their results and quality. Some of those examined a multitude of dietary habits at once that may interfere with the results. In most of those, a beneficial effect of coffee could only be observed in the groups with the highest amount of caffeine intake, which means up to 5 cups per day/a liter per day. The most significant outcomes were found in smaller studies that had a closer look at the coffee intake, including the duration of coffee consumption in years and the intake classified into many categories.

Coffee and caffeine may reduce the risk of some diseases significantly if contained in high doses what equals about six cups of coffee per day. It is still questionable if coffee intake up to almost a liter should be advised to everyone. Importantly, over usage of caffeine-containing beverages can lead to a mild physical dependence. Caffeine addiction cannot be set by certain amounts of coffee and caffeine intake. The addiction is described by the ICD10 F15.2 diagnosis: other stimulant dependence. Therefore, the same criteria, as in any other addiction must be fulfilled. A strong desire to take the substance, difficulties in controlling the intake, a physiological withdrawal state when substance use has ceased or has been reduced, evidence of tolerance, such as increased doses, progressive neglect of alternative pleasures or interest. Because of its psychological importance, caffeine withdrawal has been investigated in different studies. These studies demonstrated that the withdrawal starts 12–24 h after stopping caffeine intake and can last up to 9 days (122). People who take a minimum of 100 mg caffeine per day can acquire a physical dependence that would trigger comparatively rather mild withdrawal symptoms that include muscle pain, stiffness, lethargy, nausea, vomiting, and depressed mood (122). The U.S. Food and Drug Administration (FDA) banned some pure caffeine powders and liquids in 2015 after deaths due to caffeine overdoses were reported. Recently, the FDA banned the sale of large tubs, containing dangerous amounts of caffeine in one package. One teaspoon of this coffee powder has the equivalent amount of caffeine as 28 cups of coffee (150 ml coffee/cup) (123).

Over the last years, caffeine has also been investigated concerning Alzheimer's disease (AD) because of its potential effect as an antioxidant and by reducing oxidative stress. AD is defined as a progressive and irreversible neurodegenerative disease, which evolves into a disorder, concerning memory, cognition, and behavior. It is assumed that it affects 50% of people over the age of 80 years (124). A recent epidemiological study came to the results, that a long-term caffeine intake for more than 10 years decreases the risk of AD (125). Another 21-year follow-up study found an inverse association between coffee intake and the risk of developing AD. A moderate consumption of coffee of 3 to 5 cups daily, significantly reduces the risk of AD (62–64%) and dementia (65–70%) in the later lifetime, compared to a lower consumption of 0 to 2 cups per day (126).

Also in animal models of AD, effects of caffeine have been investigated. Chronic caffeine intake in APPsw mice had a protective effect on cognition and also reduced brain ß-amyloid production and deposits (127). Mice, which received 1,5 mg caffeine per day for 4–5 weeks which is equivalent to an intake of 500 mg/d caffeine (4–6 cups of coffee) showed a significant improvement in memory, compared to controls (128). These highly interesting results should also be examined more closely in future studies.

Coffee and caffeine may reduce the risk of some neurodegenerative diseases significantly if contained in high doses. Still the mechanisms which are responsible for the beneficial effects are not completely understood so far. In all these neurodegenerative disorders it is assumed that besides dietary habits, also genetics and additional environmental factors may contribute. In future studies, the facet of the impact of coffee and caffeine that should be investigated more closely is their effects to possibly reduce severity of disease course and of various neurological symptoms.

## Data Availability Statement

All datasets analyzed for this study are included in the manuscript and the Supplementary Files.

## Author Contributions

LH and RW outlined the subject of the review, searched for analyses, interpreted literature and wrote the manuscript. LH and RW agreed to be accountable for all aspects of the work.

### Conflict of Interest Statement

The authors declare that the research was conducted in the absence of any commercial or financial relationships that could be construed as a potential conflict of interest.

## Abbreviations

AD: Alzheimer's disease
ALS: amyotrophic lateral sclerosis
cAMP: cyclic adenosine monophosphate
CNS: central nervous system
EAE: experimental autoimmune encephalomyelitis
EDSS: expanded disability status scale
GABA: gamma-aminobutyric acid
MS: multiple sclerosis
OR: Odd's ratio
PD: Parkinson's disease
PDE: phosphodiesterase
PKA: protein kinase A.
