# Supplementary material for: The Impact of Coffee and Caffeine on Multiple Sclerosis Compared to Other Neurodegenerative Diseases
Source: Front Nutr. 2018 Dec 21;5:133. doi: 10.3389/fnut.2018.00133 (PMC6308803; doi:10.3389/fnut.2018.00133)
Supplement: Supplementary file 1 [file Data_Sheet_1.pdf]

Supplementary Material

The impact of coffee and caffeine on multiple sclerosis

A systematic review of beneficial or harmful and potential neuroprotective effects

Lena Herden, Robert Weissert*,

*** Correspondence:** Robert Weissert, MD PhD FAAN, robert.weissert@ukr.de

Supplemetary Table 1: Studies investigating the relationship between coffee or caffeine intake and Amyotrophic lateral sclerosis (ALS) and its animal model

	References	Study design	Cases	Findings
**Clinical trials**	**Fondell et al. 2015 US (78)**	Meta-analysis of five large cohort studies investigating the association between caffeine, coffee and tea and the risk of ALS	1 010 000 cases with n=1279 ALS patients during a mean 18-year follow-up<br>Mean age at baseline 59.9 ± 8.1	Caffeine intake was not associated with ALS risk, whether for men nor women, in none of the five individual studies.
	**Beghi et al 2011 Italy (79)**	Case-control study on how caffeine intake is associated with the risk of ALS	n= 485 ALS patients n= 820 controls<br>Age 27-89	Coffee intake was less frequent among patients with ALS.(p=0,0004) There was an inverse relationship between ever coffee consumption versus never consumption and the risk of ALS (OR=0,6, 95% CI 0,4-0,8)
	**Morozova et al. 2008 US (80)**	Prospective study among participants of the Cancer Prevention Study II	Cohort n=1186622<br>n= 923 ALS cases<br>Mean age: women 63, men 63.5	Consumption of decaffeinated coffee was associated with an increased risk of ALS. There were no significant results about coffee, containing caffeine
**Animal model**	**Potenza et al. 2013 Italy (81)**	Experimental study on how caffeine intake affected survival and/or motor performance in a transgenic model of ALS	$SOD1^{G93A}$ mice	Caffeine intake significantly shortened the survival of $SOD1^{G93A}$ mice and induced a nonsignificant advancing of disease onset

Supplementary Table 2: Studies examining the effect of coffee or caffeine on Parkinson's disease (PD)

	Reference	Study	Cases	Findings
**Clinical trials**	***Postuma et al. 2017 Canada (84)***	Multicenter parallel-group controlled trial on caffeine as a symptomatic treatment	n=60 caffeine group<br>Age 62.4 ± 7.5<br>n=61 placebo group<br>Age 62.3 ± 8.4	Caffeine did not provide clinically important improvement of motor manifestations of PD
	Ferreira et al. 2016 Portugal (18)	Prospective crossover placebo controlled study on the effect of espresso on daytime somnolence at PD	n=4 men<br>Age 47-75	3 blind crossover treatment periods allowed significant results. A beneficial therapeutic effect could have been shown in 50% of the patients
	Darweesh et al. 2015 Netherlands (A)	Comparing data from the first two sub cohorts of the Rotterdam Study on lifestyle factors, influencing the risk of PD	PD Cases in both studies n=94<br>Age 69.3 ± 9.1 and<br>Age 64.5 ± 7.9	No significant association between coffee consumption and the risk of developing PD could have been observed
	Kumar et al. 2015 Singapore (96)	Case-control study comparing caffeine intake effects on genetic high- and low-susceptibility to PD	812 subjects<br>n=378 cases with PD<br>n= 434 controls<br>Age 25-90	Caffeine intake significantly reduces the risk of PD much more in those with high genetic susceptibility to PD compared to those with low genetic susceptibility to PD
	Van der Mark et al. 2014 Netherlands (97)	Case-control study to investigate the possible risk of alcohol, coffee or smoking on PD	PD cases n=444, Controls n=876<br>Age 34-91	Results observed some indication of a potential inverse relation between total and average coffee consumption and PD
	Qi and Li 2014 China (B)	A dose-response meta-analysis on coffee and caffeine consumption	n=492722 in coffee group and n=901764 in caffeine group	Coffee and caffeine consumption have inverse associations with the risk of developing PD<br>Coffee at three cups/d (volume not identified): reduced risk of PD (RR=0.72, CI=0.65-0.81)
	Palacios et al. 2012 US (87)	Prospective study on caffeine intake and risk of PD within the Cancer Prevention Study II Nutrition Cohort	n=63590 women and n= 48532 men with an 8 year follow up<br>Mean age 71 (men) and 69 (women)	Caffeine has a protective effect against the risk of developing PD<br>**Men:** Caffeine at ≥274 mg/d (≥2 cups coffee/d) reduces risk of PD by 50% (RR=0.54, CI=0.37-0.80) vs 9.2 mg/d<br>Caffeine at 478 mg/d reduces risk of PD (RR=0.43, CI=0.26-0.71) vs 9.2 mg/d<br>**Women:** Caffeine at 435 mg/d (3.2 cups coffee/d) reduces risk of PD by 40% (RR=0.61, CI=0.34-1.09) vs 5.6 mg/d.
	Liu et al. 2012 US (88)	Prospective study concerning whether caffeine intake or smoking is associated with risk of PD	n=318260 participants (61 year)<br>9-11 years follow up	Caffeine has an inverse association with the risk of developing PD<br>Coffee at >5 cups/d: reduced risk of PD in men (OR=0.70, CI=0.47-1.04) and women (OR=0.74, CI=0.42-1.29) vs nonusers
	Postuma et al. 2012 Canada (83)	6-week randomized controlled trial of caffeine in PD. Primary outcome was the Epworth sleepiness scale (ESS)	n= 31 placebo group<br>Age 67.8± 11.2<br>n=30 caffeine group<br>Age 65.2 ± 8.3	Caffeine treatment in PD patients has potential motor benefits and improved total UPDRS (United Parkinson Disease Rating Scale) by 4.7 points. There were no significant changes in the ESS-score.
	Kandinov et al. 2009 Israel (112)	Retrospective analysis on the effect of coffee and tea on the onset of PD	n= 278 PD patients	Coffee consumption exceeding 3 cups per day advanced the age of PD onset by 4.8 years (p=0.03)

Simon et al. 2008 US (85)	Analysis of the data of two studies, evaluating the relationship between caffeine intake and the rate of progression of PD	n=413 early PD cases<br>Age: patients older than 30 were included	Rate of progression of PD did not differ significantly between those in the highest and lowest quartiles for caffeine
Facheris et al. 2008 US (98)	Case-control study evaluating how genetic factors contribute to the effect of coffee on PD	n=1208<br>446 case-unaffected sibling pairs and 158 case-unrelated control pairs<br>Age 31-87	No significant association between the ADORA2A, the CYP1A2 gene and the effect of coffee on PD could have been shown
Powers et al. 2008 US (99)	Case-control study to examine the association between smoking, coffee and NSAIDs and PD	Cases n=1186, Controls n=928<br>Age 25-97	A dose-dependent inverse relation between coffee intake and the risk PD was shown with higher significance in man than women
Hu et al. 2007 Finland (89)	Prospective study on coffee and tea consumption and the risk of PD	n=15042 women n=14293 men<br>200 cases had a<br>12,9 years of follow-up<br>Age 25-74	Coffee drinking is associated with lower risk of developing PD<br>**Men**:0, 1-4 cups, and >5 cups of coffee (100 mL/cup) had a hazard ratio of 1.00, 0.55 (CI=0.26-1.15) and 0.41 (CI=0.19-0.88), respectively, of PD<br>**Women**: 0, 1-4 cups and >5 cups of coffee (100 mL/cup) had a hazard ratio of 1.00, 0.50 (CI=0.22-1.12) and 0.39 (CI=0.17-0.89), respectively, for PD
Sääksjärvi et al. 2007 Finland (90)	Prospective study to examine the prediction if coffee consumption on the incidence of PD	Cohort of n= 6710 participants, after a 22-year follow-up PD cases were n=101<br>Age 30-75	Results suggest that high coffee consumption (< 10 cups/day) lead to reduction of risk for PD, but the protective effect of coffee may vary by exposure to other factors
Kitagawa et al. 2007 Japan (86)	Prospective study, evaluating the effect of 100mg caffeine on freeze of gait (FOG) in PD	n=16 patients<br>Age 56 -82	Caffeine improved "total akinesia" type of FOG, but had no effect on "trembling in place." Tolerance developed to the beneficial effect of caffeine on FOG within a few months, but a 2-week caffeine withdrawal period could restore the effect of caffeine
Hancock et al. 2007 US (100)	Family-based Case-Control study to examine the effects of coffee, tea and soft drinks on PD	Cases n=356<br>Controls n=317, matched in age and sex<br>Age 66.1 $\pm$ 10.7	Inverse associations of smoking and caffeine were shown. More than 2 cups/day were significantly inversely associated with PD (OR=0.64)
Evans 2006 UK (101)	Case-Control study to examine the effect of coffee, tea, chocolate milk and soft drinks on PD	Cases n=150<br>Controls n=150, matched in age and sex<br>Age 38-81	Higher caffeine intake was associated with lower risk of PD (OR=0.74, p=0.007)
Wirdefeldt et al. 2005 Sweden (102)	Case-control study assessing the association between lifestyle factors and the risk of PD in a co-twin control study	PD cases n= 476, Controls n= 2380<br>(415 same sex twin pairs)<br>Mean age 74.9 $\pm$ 8.6	No association between coffee and PD could have been observed.
Ascherio et al. 2004 US (92)	Statistical analysis concerning the coffee intake and estrogen use	n=301.164 men and n=238.058 women 909 men and 349 women with a 10 year follow up<br>Mean age 57(men) and 56 (women)	Consumption of caffeinated coffee was associated with a reduced PD mortality among men and among women who never used postmenopausal estrogens.

Ascherio et al. 2003 US (91)	Statistical analysis concerning the coffee intake and estrogen use	n=77713 women n=154 women with a 18 year follow up Age 30-55	Use of postmenopausal hormones was associated with a lower risk of PD among women with low caffeine intake, but with an increased risk among women with high caffeine intake
Tan et al. 2003 Singapore (103)	Case-control study to examine the relationship between coffee and tea intake, smoking and environmental factors and the risk of PD	Cases n=200 Controls n=200, matched in age and sex Age 43-88	A dose-dependent protective effect of PD could have been demonstrated for coffee (OR= 0.79, p=0.006)
Ragonese et al. 2003 Italy (104)	Case-control study investigating the association between smoking, alcohol, coffee intake and PD	Cases n=150 Controls n=150, matched in age and sex Age 31-81	Results suggest a strong inverse association between coffee drinking an PD, in ever versus never coffee drinkers were evaluated (OR= 0.16, p=0.0001)
Checkoway et al. 2002 US (105)	Case-control study, observing the relationship between smoking, alcohol and caffeine intake and the risk of PD	n=210 cases and n=347 controls matched in age and sex Age 37-88	No association between risk of PD and coffee consumption or total caffeine intake
Paganini-Hill 2001 US (106)	Case-control study assessing the risk factors for PD	n=395 PD cases 2320 controls Mean age 75±6,1	The risk of PD was significantly reduced among coffee drinkers, who drank at least 2 cups of coffee/day (OR= 0.64)
Ascherio 2001 US (93)	Prospective cohort study evaluating the effect of coffee on the risk for PD	n=47351 men and n=88565 women, n=157 men 10 year follow up. n=131 women 16 year follow up, Age 40-75	Highly significant inverse association between caffeine intake and risk of PD among men (p=0.001)
Ross et al. 2000 US (94)	Prospective cohort study concerning the association between coffee and caffeine intake and PD	n=8004 men with 102 cases with 30 years follow up Age 45-68	Caffeine has an inverse association with the risk of developing PD. More than 421 mg of caffeine/d is associated with 5 times lower risk of developing PD vs nondrinkers (p<0.001)
Benedetti et al. 2000 US (107)	Case-control study on the association between coffee intake and smoking on PD	n=202 PD patients n=202 controls Age 41-97	Coffee was more common in controls than in cases (OR=0.35)
Preux et al. 2000 France (108)	Case-control study assessing environmental factors as risk for PD	n=140 PD patients n=280 controls Mean age 71.1 ± 7.5	No association between coffee intake and risk of PD
Hern et al. 2000 (95)	Prospective cohort study on the possible protective effect of coffee for PD	n= 8004 , n= 102 cases of PD during a 27-year follow-up, Average age 53	A dose-response relationship was observed. Higher amounts of coffee intake were associated with lower risk of developing PD
Fall 1999 Sweden (109)	Case-control study on nutritional factors influencing the risk of PD	n=113 PD patients n=263 controls Age 30-86	No significant association has been found
Hellenbrand 1996 Germany (110)	Case-control study to examine the association between coffee, tea and the risk of PD	n=342 cases n=342 controls Mean age 56.2±6.7 (cases), 56.1 ±6.9 (controls)	Cases consumed less coffee (OR= 0.27, p=0.0003, highest versus lowest quartile) than controls
Morano 1994 Spain (111)	Case-control study on risk factors for PD	n=74 PD patients n=148 controls	No association between coffee drinking habits and risk of PD

	Nefzger et al 1968 US (C)	Retrospective study on smoking and coffee consumption	n=198 PD patients<br>n=198 other patients	No association between coffee consumption and onset of PD.

Supplementary Table 3: Studies examining the effect of coffee and/or caffeine in animal model of PD

**Animal model**	**Bagga et al. 2015 US (117)**	Evaluation of the neuroprotective effect in the MPTP model of PD by monitoring brain regions	C57BL5 mice (male, 4 months)<br>n=10 control<br>n=7 caffeine<br>n=10 MPTP<br>n=8 MPTP and caffeine	Caffeine reduces neuron damage in the striatum Caffeine increases motor function (60.6% improvement in grip strength). Pretreatment with caffeine provides partial neuroprotection against severe striatal degeneration in PD.
	**Sonsalla et al. 2012 US (56)**	Experimental study investigating chronic caffeine treatment of rat brains	Sprague Dawley rats<br>n=5 per group<br>Males Age not identified	Caffeine reduces loss of nigral dopamine cell bodies at 1st week or 3rd week by 94% and 69%, respectively. Caffeine reduces microglia activation in the substantia nigra and protects against the loss of nigral dopamine neurons in a chronic progressive rat model of PD.
	**Nakaso et al. 2008 Japan (120)**	Experimental study investigating cytoprotective mechanisms of caffeine	Human dopaminergic neuroblastoma cell lines	Caffeine reduces cell death and reduces the number of apoptotic nuclei from 13.1% to 9.7% under MPP+-exposed conditions<br>Caffeine reduces caspase 3 in a dose-dependent manner by 21%
	**Xu et al. 2006 US (116)**	Experimental study on the correlation between estrogen and caffeine, concerning the neuroprotective effects of caffeine	C57BL6 mice<br>n=3-7 saline treatment<br>n=4-15 MPTP<br>Young: 10 week<br>Old: 6-9 months	Estrogen can prevent the neuroprotective effects of caffeine in a model of PD mice Caffeine reduces MPTP-induced dopamine loss in a dose-dependent manner in male mice, with maximal effects achieved at 10 mg/kg.<br>In ovariectomized mice treated with estrogen, caffeine was neuroprotective only at higher doses (40 mg/kg).
	**Joghataie 2004 Iran (118)**	Experimental study on the effect of caffeine to the brain of wistar rats.	Adult male Wistar rats<br>n=72<br>18 per group	Caffeine administration for 1 month could attenuate the rotational behavior in lesioned rats and protect the neurons of SNC against 6-OHDA toxicity Nigrostriatal neurons within SNC were mainly preserved against neurodegenerative effects induced by the neurotoxin 6-OHDA
	**Chen et al. 2001 US (114)**	Experimental study on the neuroprotective effects of caffeine and the inactivation of adenosine receptors	C57BL6 mice (male 9 months)<br>n=13 MPTP<br>n=5 saline	Caffeine (10 mg/kg): residual dopamine was 40% of control vs 15% of Control<br>Caffeine (20 mg/kg): reversed MPTP-induced dopamine depletion Caffeine at higher dosage caused excessive systemic toxicity

Additional references

A. Darweesh SK, Koudstaal PJ, Stricker BH, Hofman A, Ikram MA. Trends in the Incidence of Parkinson Disease in the General Population. *Am J Epidemiol* (2016) **183**(11):1018–26. doi:10.1093/aje/kwv271

B. Qi H, Li S. Dose-response meta-analysis on coffee, tea and caffeine consumption with risk of Parkinson's disease. *Geriatr Gerontol Int* (2014) **14**(2):430–9. doi:10.1111/ggi.12123

C. Nefzger D, Quadfasel FA, Karl VC. A retrospective study of smoking in parkinsons disease. *Am J Epidemiol* (1968) **88**(2):149–58.

Research Strategy

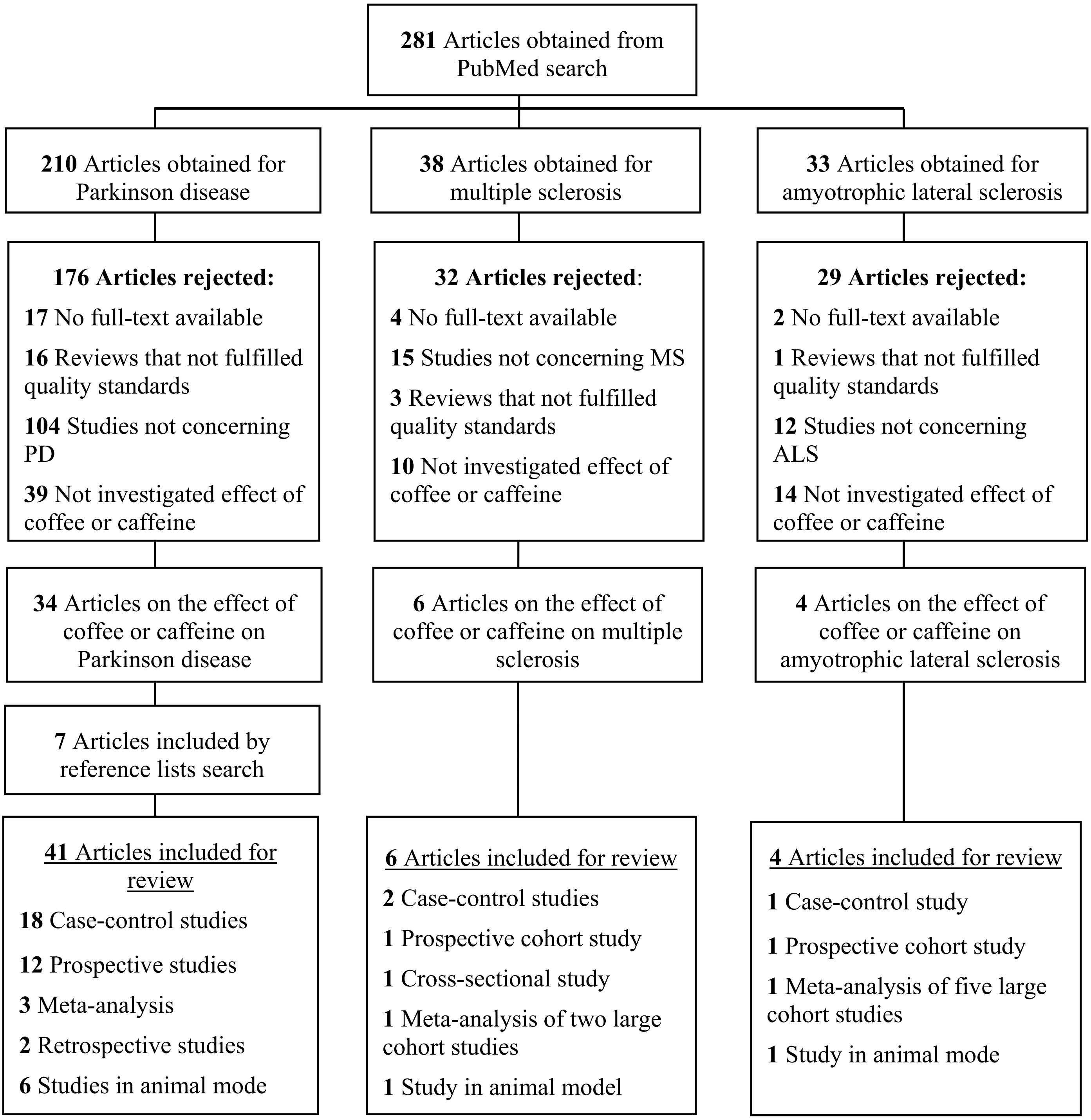

Supplementary Figure 1: Flow diagram of the study selection process.
